# 2-thio-6-azauridine inhibits Vpu mediated BST-2 degradation

**DOI:** 10.1186/s12977-016-0247-z

**Published:** 2016-03-02

**Authors:** Quan Zhang, Zeyun Mi, Yuming Huang, Ling Ma, Jiwei Ding, Jing Wang, Yongxin Zhang, Yang chen, Jinming Zhou, Fei Guo, Xiaoyu Li, Shan Cen

**Affiliations:** Institute of Medicinal Biotechnology, Chinese Academy of Medical Sciences and Peking Union Medical College, Beijing, 100050 China; Beijing Ditan Hospital, Capital Medical University, Beijing, China; Institute of Pathogen Biology, Chinese Academy of Medical Sciences and Peking Union Medical College, Beijing, China; Department of Biochemistry and Molecular Biology, Tianjin Medical University, Tianjin, China

**Keywords:** HIV-1, BST-2, Vpu, Membrane protein, 2-thio-6-azauridine, β-TrCP2, Ubiquitination

## Abstract

**Backgroud:**

BST-2 is an interferon-induced host restriction factor that inhibits the release of diverse mammalian enveloped viruses from infected cells by physically trapping the newly formed virions onto the host cell surface. Human Immunodeficiency Virus-1 (HIV-1) encodes an accessory protein Vpu that antagonizes BST-2 by down-regulating BST-2 from the cell surface.

**Results:**

Using a cell-based ELISA screening system, we have discovered a lead compound, 2-thio-6-azauridine, that restores cell surface BST-2 level in the presence of Vpu. This compound has no effect on the expression of BST-2 and Vpu, but inhibits Vpu-mediated BST-2 down-regulation and exerts no effect on Vpu-induced down-regulation of CD4 or KSHV K5 protein induced BST-2 down-regulation. 2-thio-6-azauridine suppresses HIV-1 production in a BST-2-dependent manner. Further results indicate that 2-thio-6-azauridine does not interrupt the interaction of BST-2 with Vpu and β-TrCP2, but decreases BST-2 ubiquitination.

**Conclusion:**

Our study demonstrates the feasibility of using small molecules to target Vpu function and sensitize wild type HIV-1 to BST-2-mediated host restriction.

**Electronic supplementary material:**

The online version of this article (doi:10.1186/s12977-016-0247-z) contains supplementary material, which is available to authorized users.

## Background

Human bone marrow stromal cell antigen 2 (BST-2, also called Tetherin, CD317 or HM1.24) was first shown to inhibit HIV-1 production by physically trapping the newly formed virions onto the host cell surface [1, 2]. Its antiviral spectrum was subsequently expanded to other enveloped viruses including retroviruses, arenaviruses, herpesviruses, and filoviruses [2–9]. BST-2 is a 28- to 36-kDa type II integral membrane glycoprotein, located to lipid rafts at the plasma membrane and the trans Golgi network (TGN). It consists of four domains, a short N-terminal cytoplasmic tail, an N-terminal transmembrane region (TMR), an extracellular coiled-coil domain and a C-terminal glycosyl-phosphatidylinositol (GPI) anchor [10–12]. The unique topology that is determined by the GPI anchor and the coiled-coil structure, rather than by the amino acid sequence, confers its virus-tethering activity [1, 13].

Many viruses have evolved countermeasures to overcome BST-2 restriction [4, 14–20]. In HIV-1, a 16-kDa accessory protein Vpu fulfills this role. Vpu is also an integral membrane protein, which belongs to type I integral membrane protein family. It performs two distinct functions in HIV-1 infection: causing CD4 degradation in the endoplasmic reticulum and promoting viral particle release by counteracting BST-2 [21–28]. The mechanisms underlying Vpu’s antagonistic activity against BST-2 involve a direct interaction between their respective transmembrane (TM) domains, targeting BST-2 to beta Transducin-repeat Containing Protein2 (β-TrCP2) dependent lysosomal and/or proteasomal degradation pathway [14, 29–31], or simply trapping BST-2 within the TGN [32, 33]. Since studies have demonstrated decreases in levels of both the total cellular BST-2 as well as the cell surface BST-2 by Vpu [29, 34–37], both the lysosomal and the proteasomal degradation pathways may have a role in Vpu antagonization of BST-2.

BST-2 is constitutively expressed in many human cell types including B cells, T cells, monocytes, macrophages, plasmacytoid dendritic cells (PDCs) as well as cell lines such as HeLa, H9 and Jurkat [1, 34, 38]. Since the natural HIV-1 target cells are among the above list, blocking Vpu-mediated BST-2 down-regulation represents an attractive strategy for developing new anti-HIV drugs. In the previous study, we have developed a high-throughput cell-based ELISA assay to monitor cell surface BST-2 level. This assay has been successfully utilized to identify small molecules that antagonize HIV-1 Vpu function and thus inhibit HIV-1 production by rescuing the antiviral activity of BST-2 [39].

## Results

### 2-thio-6-azauridine inhibits Vpu-mediated down-regulation of cell surface BST-2

**Fig. 1:**
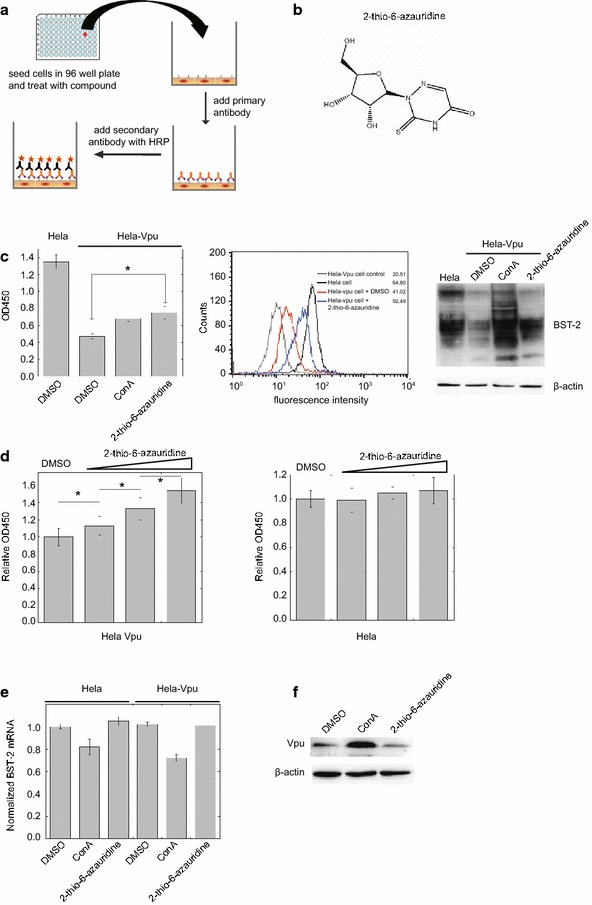
2-thio-6-azauridine inhibits Vpu-mediated down-regulation of cell surface BST-2. **a** Illustration of the assay that was used to screen small molecules that antagonize HIV-1 Vpu function and inhibit BST-2 degradation. **b** Structure of 2-thio-6-azauridine. **c** HeLa and HeLa-Vpu cells were treated by 2-thio-6-azauridine for 24 h. Levels of cell surface BST-2 were detected using cell-ELISA (*left panel*), flow cytometry (*middle pannel*) or Western blotting (*right panel*) (**d**) HeLa-Vpu and HeLa cells were treated with increasing concentrations of 2-thio-6-azauridine (0.05, 0.5, 5 µM) for 24 h. Cell surface BST-2 was measured using cell-ELISA. (**e**) HeLa and HeLa-Vpu cells were treated with DMSO, 2-thio-6-azauridine (5 µM) and ConA (50 nM) for 24 h respectively. The amounts of *bst*-*2* mRNA were measured by real-time RT-PCR. The *bst*-*2* mRNA values are normalized to those in the control cells that were treated with DMSO. **f** HeLa-Vpu cells were treated with DMSO, 2-thio-6-azauridine (5 µM) and ConA (50 nM) for 24 h respectively. Western blots of cell lysates were probed with anti-Vpu (*top panel*) and anti-β-actin (*bottom panel*) antibodies, respectively. (*p < 0.05, t test)

We have previously developed a high-throughput cell-based ELISA assay for screening small molecules that prevent HIV-1 Vpu from degrading BST-2 [39]. Briefly, we firstly established a stable Vpu-expressing HeLa cell line, HeLa-Vpu, which continuously expresses Vpu and down-regulates the BST-2 level on the cell surface. Next, we fixed the cells in 96-well microplates and monitored the cell surface BST-2 level using enzyme-linked Cell-ELISA. After optimization and evaluation, the assay model was adapted to a cell-based high-throughput screening (HTS) assay to identify compounds that inhibit Vpu-mediated BST-2 degradation (Fig. 1a).

Using this assay, we performed a screening test against 56,000 compounds in the chemical library and natural product collection at the Institute of Medicinal Biotechnology at a concentration of 10 μM. A positive hit was defined a priori as a compound that would increase cell ELISA optical density at OD450 by more than 3 standard deviations (SD) in the primary screen. Positive hits were then tested further to exclude a possible false-positive effect due to optical density of compound. Finally, the positive compounds were tested for cellular toxicity effects, dose–response assessments and anti-HIV activities. In the end, three compounds including 2-thio-6- azauridine (MW 261.26, Fig. 1b) were identified to be effective in restoring cell ELISA optical density at OD450, the value that reflects the cell surface level of BST-2. Previous reports and our data showed that 2-thio-6-azauridine was a low toxicity compound with antitumor and antivirus activity [40, 41]; we therefore chose 2-thio-6-azauridine for further mechanism investigation. Initial results revealed that 2-thio-6-azauridine is capable of restoring BST-2 level on the cell surface, which were determined using the cell ELISA and a FACS analysis (Fig. 1c, left and middle panels). Results of Western blots confirmed that 2-thio-6-azauridine can significantly restore BST-2 expression in the presence of HIV-1 Vpu, a property that is shared by the lysosomal inhibitor Concanamycin A (ConA) (Fig. 1c, right panel).

In order to determine whether this effect of 2-thio-6-azauridine is specific to Vpu, we treated HeLa-Vpu and HeLa cells with increasing concentrations of 2-thio-6-azauridine (0.05, 0.5, 5 µM) respectively for 24 h, and then measured the cell surface levels of BST-2 by cell-ELISA. A dose-dependent increase of the BST-2 levels was observed only in HeLa-Vpu cells, not in HeLa cells (Fig. 1d). The similar results were obtained using the FACS analysis (Additional file 1). These data suggest that 2-thio-6-azauridine increases the cell surface BST-2 not simply by up-regulating the expression of BST-2. We also measured the bst-2 mRNA level in HeLa-Vpu and HeLa cells using real-time RT-PCR. The results showed that the compound had nearly no effect on bst-2 mRNA level in both cell lines, which further confirms that 2-thio-6-azauridine does not affect BST-2 expression (Fig. 1e). We next examined the possibility whether 2-thio-6-azauridine affects Vpu expression. The results of Western blotting showed no effect of 2-thio-6-azauridine on Vpu level in the HeLa-Vpu cells (Fig. 1f). Taken altogether, our results indicate that 2-thio-6-azauridine does not affect the expression of BST-2 or Vpu, which suggests that 2-thio-6-azauridine restores the level of cell surface BST-2 by inhibiting Vpu-mediated BST-2 degradation.

### 2-thio-6-azauridine inhibits HIV-1 production in a BST-2 dependent manner

**Fig. 2:**
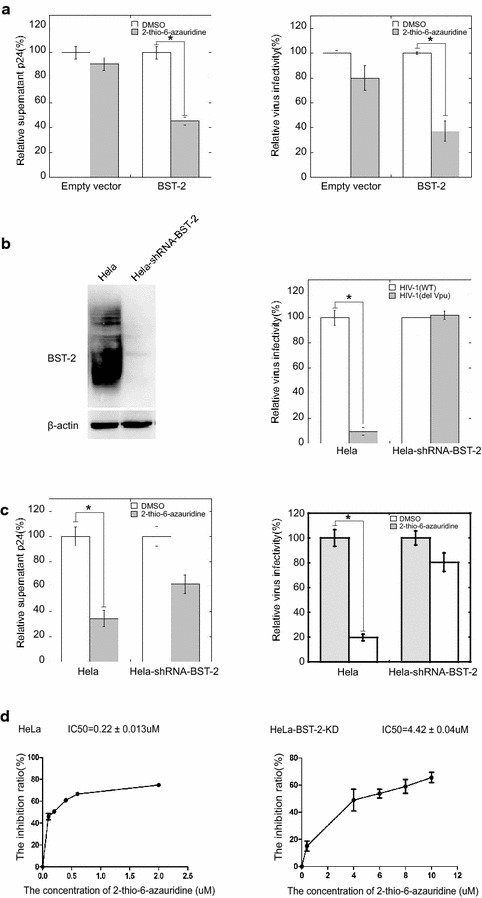
2-thio-6-azauridine inhibits HIV-1 in a BST-2-dependent manner. **a** Single-round pseudotyped HIV-1 expressing plasmids pNL-Luc-E- and pHIT/G were co-transfected with pBST-2 which expresses HA tagged BST-2 or the control vector to 293T cells. 24 h post transfection, the cells were treated with 5 μM of 2-thio-6-azauridine. Viruses in the culture supernatants were harvested at 48 h post transfection. Viral p24 and virus infectivity were measured as described in “Methods”. **b** Establishment of HeLa-shRNA-BST-2 cells, a BST-2 knockdown HeLa cell line. Western blot detection of BST-2 expression in HeLa and HeLa-shRNA-BST-2 cells is shown in the *left panel*. Results of relative infectivity assay using a single-round HIV-1 infection in HeLa and HeLa-shRNA-BST-2 cells are shown in the right panel. **c** HeLa and HeLa-shRNA-BST-2 cells were co-transfected with plasmids pNL-Luc-E- and pHIT/G to produce single-round HIV-1 particles. Cells were treated with 5 μM of 2-thio-6-azauridine 24 h post transfection. Viral p24 and virus infectivity measured as described in “Methods”. **d** The IC50 value of 2-thio-6-azauridine in HeLa and HeLa-shRNA-BST-2 cells. (*p < 0.05, t test)

Since 2-thio-6-azauridine prevents Vpu from down-regulating BST-2, we next tested whether 2-thio-6-azauridine inhibits the production of pseudotyped HIV-1 in the presence of BST-2. Plasmids pNL-Luc-E- and pHIT/G were co-transfected with either pBST-2 or the control vector into 293T cells that do not express endogenous BST-2. Cells were treated with 5 μM of 2-thio-6-azauridine at 24 h post transfection. Viruses in the culture supernatant were harvested at 48 h post transfection. The amounts of viruses were determined by measuring p24 in the supernatant (Additional file 2A). Equal amounts of viruses were used to infect SupT1 cells to determine virus infectivity. As shown in Fig. 2a, 2-thio-6-azauridine treatment reduced virus amount in the supernatant by approximately 60 % in the presence of BST-2, whereas no significant effect was seen in the absence of BST-2. A 60 % decrease was detected in virus infectivity in the presence of BST-2. These data suggest that 2-thio-6-azauridine sensitizes the wild type HIV-1 to BST-2 restriction by preventing Vpu from countering BST-2.

We next tested the effect of 2-thio-6-azauridine on the antiviral activity of endogenous BST-2. To this end, a BST-2 knockdown HeLa cell line, named HeLa-shRNA-BST-2, was generated. Results of Western blotting showed that this cell line expressed almost no BST-2 (Fig. 2b, left panel). As a result, this cell line produced 7 to eightfold more Vpu-deficient HIV-1 than the control HeLa cells (Fig. 2b, right panel). 2-thio-6-azauridine reduced the HIV-1 release and infectivity by approximately threefold in HeLa cells with an approximate IC50 value of 0.22 ± 0.013 μM. On the other hand, 2-thio-6-azauridine only showed a marginal inhibition effect against HIV-1 in HeLa-shRNA-BST-2 cells (IC50: 4.42 ± 0.004 μM, Fig. 2c, d, Additional file 2B). These results demonstrate that 2-thio-6-azauridine inhibits HIV-1 replication mainly through protecting BST-2 from Vpu-induced degradation.

**Fig. 3:**
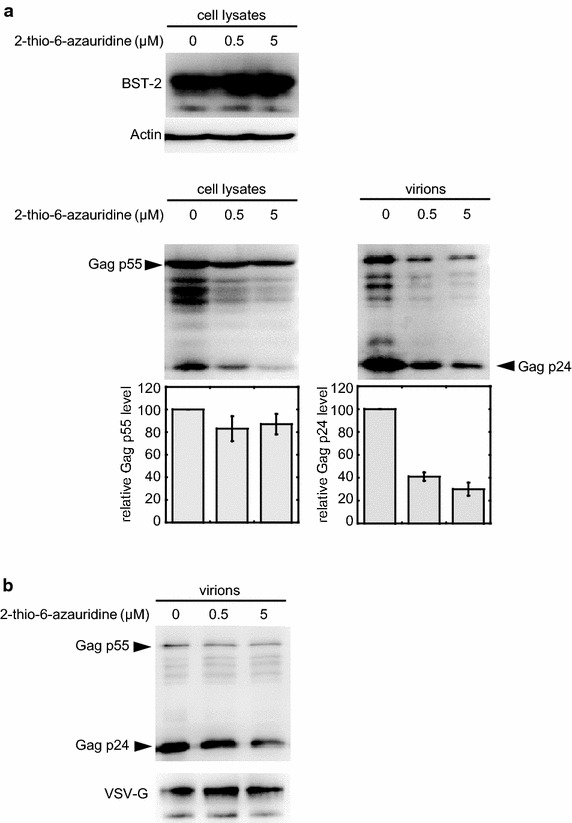
2-thio-6-azauridine reduces HIV-1 particle release. **a** Plasmids pNL-Luc-E- and pHIT/G were co-transfected with either pBST-2 or the control vector into 293T cells. Cells were treated with DMSO or different concentration of 2-thio-6-azauridine (0.05, 0.5, 5 μM) at 24 h post transfection. The cells and viruses in the culture supernatant were harvested separately at 48 h post transfection and then the HIV-1 Gag p55 in cell lysate and HIV-1 Gag P24 in virions were detected by Western Blot. **b** By normalizing the Gag p55 level in virions, the P24 and VSV-G were detected by Western Blot

To further validate the inhibitory effect of 2-thio-6-azauridine upon the virus release in the presence of BST-2, we performed a standard HIV particle release assay by western blotting. This assay involves quantitating the ratio of virion-associated Gag p24 in the supernatant relative to cell-associated Gag p55 in the cell lysates. As shown in Fig. 3a, 2-thio-6-azauridine only showed a marginal inhibition effect (less than 20 %) on HIV-1 Gag expression, while virion-associated p24 was reduced by more than 60 %. This result is consistent with that of p24 quantification using the ELISA (Fig. 2a).

It is also noteworthy that the reduction in viral infectivity by 2-thio-6-azauridine is more significant than that of virus production, suggesting that in addition to restoring BST2 levels at the cell surface and thereby impeding the release of HIV-1 particles, the compound may affect another step in the virus life cycle. Therefore, we further examined the effect of 2-thio-6-azauridine on Gag processing and VSVg pseudotyping. Interestingly, with increasing amount of 2-thio-6-azauridine, we observed gradual increase in the ratio of immature viral protein (including Gag p55 and its immature derivatives) relative to mature viral protein p24 (Fig. 3b). This suggests that the compound impairs viral Gag processing. A previous work showed that BST-2 somehow interferes with the activation of viral protease [42]. Taken together, these data suggest that restored BST-2 levels by the compound inhibit viral release and consequently impede maturation of HIV-1 particles.

**Fig. 4:**
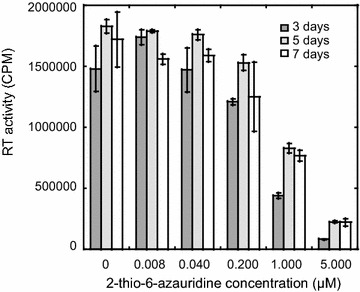
2-thio-6-azauridine reduces the infectivity of wild type HIV-1. CBMC were treated with AZ in different concentrations (0.008, 0.04, 0.2, 1,5 μM) and infected with equal amount of NL4-3 viruses. Cell supernatants were harvested on day 3, day 5, day 7 and then analyzed using an RT assay

We next examined the inhibitory effect of 2-thio-6-azauridine on the replication of wild type HIV-1 in human primary mononuclear cells. The equal amounts of cord blood mononuclear cells (CBMCs) were infected with fully infectious wild type HIV-1 strain NL4-3, followed by measuring viral reverse transcriptase activity to determine the amounts of virus in culture supernatants. The compound showed potent activity against the replication of wild type HIV-1 in human primary cells (Fig. 4).

### 2-thio-6-azauridine specifically inhibits Vpu-induced degradation of BST-2

**Fig. 5:**
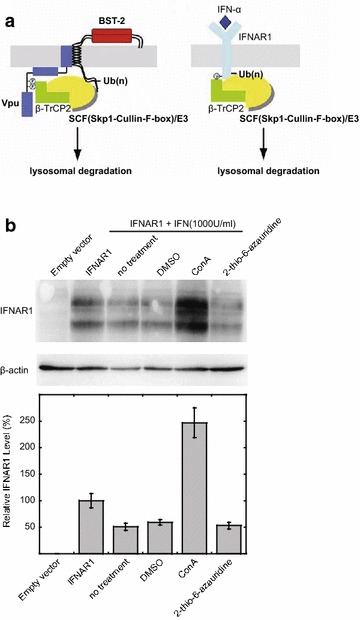
2-thio-6-azauridine dose not affect lysosomal-dependent degradation. **a** Models for the ubiquitin-mediated down-regulation of BST-2 by Vpu and for the ubiquitin-mediated down-regulation of IFNAR1 upon treatment of cells with IFN-α. *Left panel* Vpu interacts with BST-2 via the transmembrane domain and then induces BST-2 ubiquitination through the recruitment of β-TrCP2-containing E3 ubiquitin ligase complex, thus targeting the ubiquitinated proteins to lysosomal degradation. *Right panel* Treatment with IFN-α promotes phosphorylation of IFNAR1. Phosphorylated IFNAR1 recruits β-TrCP2-containing E3 ubiquitin ligase complex and is ubiquitinated, then ubiquitinated proteins are degraded through lysosomal pathway. **b** Effect of 2-thio-6-azauridine on IFN-induced down-regulation of IFNAR1. HeLa cells were transfected with IFNAR1 expressing plasmid pcDNA3-IFNAR1-FLAG or empty vector as indicated. 40 h later, cells were treated as indicated for 4 h. Western blots of cell lysates were probed with anti-FLAG (*top panel*) and anti-β-actin (*bottom panel*) antibodies, respectively

We next asked whether 2-thio-6-azauridine specifically inhibits Vpu-induced degradation of BST-2. To answer this question, we first tested the effect of 2-thio-6-azauridine on IFN-α-triggered degradation of IFN-α receptor 1 (IFNAR1). IFNAR1 is a type I membrane protein. Interferon stimulation leads to IFNAR1 phosphorylation followed by β-TrCP2-dependent lysosomal degradation. This degradation model is similar to Vpu-induced BST-2 degradation (Fig. 5a) [43]. HeLa cells were first transfected with a plasmid expressing Flag-tagged IFNAR1. Then, cells were treated with actidione 40 h post transfection to block IFNAR1 synthesis. IFN-α was added to cells to induce lysosomal degradation of IFNAR1. 2-thio-6-azauridine was also added in the presence of IFN-α. As shown in Fig. 5b, IFN-α reduced IFNAR1 level and the lysosomal inhibitor ConA restored IFNAR1 expression. By contrast, 2-thio-6-azauridine had no effect on the IFN-induced IFNAR1 degradation. These data suggest that 2-thio-6-azauridine is specific in preventing Vpu-induced BST-2 degradation and has no general effect on the lysosomal degradation pathway.

**Fig. 6:**
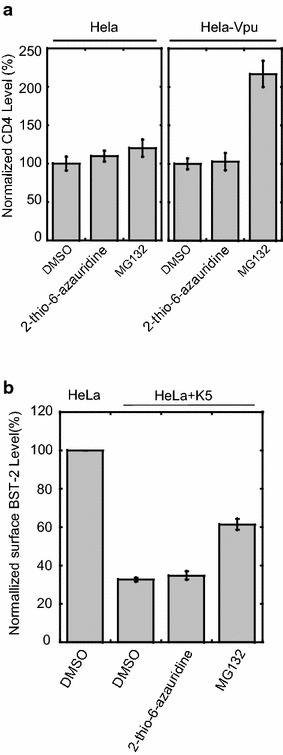
2-thio-6-azauridine dose not affect Vpu induced down-regulation of cell surface CD4 and K5 induced BST-2 degradation. **a** pMX hCD4 plasmid expressing human CD4 was transfected to HeLa or HeLa-Vpu cells, followed by treating the cells with 5 μM of 2-thio-6-azauridine at 24 h post transfection. The CD4-positive cells were scored by flow cytometry at 48 h post transfection. **b** KSHV K5 protein expressing plasmid pcDNA3.1-K5-HA was transfected to HeLa, followed by treating the cells with 5 μM of 2-thio-6-azauridine or 50 nM MG132 at 24 h post transfection. The cell surface BST-2 level was scored by flow cytometry at 48 h post transfection

In order to further demonstrate this specificity of 2-thio-6-azauridine toward Vpu/BST-2, we examined the effect of 2-thio-6-azauridine on Vpu-mediated degradation of CD4. It is known that Vpu down-regulates CD4 in the endoplasmic reticulum through β-TrCP-dependent degradation, and the treatment with proteasome inhibitor MG132 blocks the degradation process. Plasmid expressing CD4 was first transfected to HeLa or HeLa-Vpu cells, followed by treating the cells with 5 μM of 2-thio-6-azauridine at 24 h post transfection. The CD4-positive cells were scored by flow cytometry at 48 h post transfection (Additional file 3A). As shown in Fig. 6a, CD4 levels in the presence of Vpu were almost unchanged with the treatment of 2-thio-6-azauridine, while the addition of MG132 significantly increased the CD4 content. By contrast, either 2-thio-6-azauridine or MG132 exhibited no significant effect upon CD4 in the absence of Vpu. The results suggest that 2-thio-6-azauridine specifically inhibits Vpu-induced BST-2 degradation and has no inhibitory effect to other function of Vpu protein.

BST-2 has been shown to inhibit the release of a variety of enveloped viruses, such as HIV-2, Simian Immunodeficiency Viruses, Kaposi’s sarcoma- associated herpesvirus (KSHV), etc. [3, 4, 18, 20, 44]. These viruses have also developed various mechanisms to counteract the activity of BST-2. For example, KSHV encodes a RING-CH E3 ubiquitin ligase K5, which can antagonize BST-2 in a similar way as Vpu [20]. To examine whether 2-thio-6-azauridine inhibits the degradation of BST2 by K5, plasmid expressing K5 was transfected to HeLa cells, followed by treating the cells with 5 μM of 2-thio-6-azauridine or 50 nM MG132 at 24 h post transfection. The surface BST-2 level of cells was scored by flow cytometry at 48 h post transfection (Additional file 3B). As shown in Fig. 6b, the treatment of 2-thio-6-azauridine did not restore the cell surface BST-2 level reduced by K5. These results demonstrate that 2-thio-6-azauridine has no inhibitory effect upon K5 induced BST-2 degradation. Together, these data suggest that 2-thio-6-azauridine specifically blocks Vpu-induced degradation of BST-2.

### 2-thio-6-azauridine does not affect the interaction of BST-2 with Vpu

**Fig. 7:**
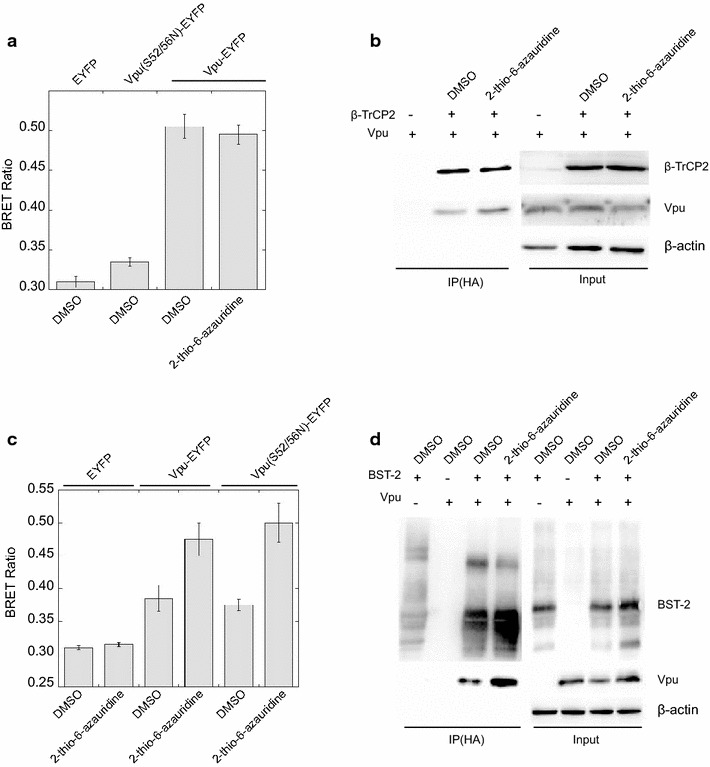
Effect of 2-thio-6-azauridine on BST-2/Vpu/β-TrCP2 interaction. **a** Effect of 2-thio-6-azauridine on the interaction between Vpu and β-TrCP2. 293T cells were co-transfected with pEYFP-N1-Vpu, pRluc-C3-β-TrCP2 and pBST-2, which express EYFP tagged Vpu, RLuc tagged β-TrCP2 and HA tagged BST-2 respectively. 24 h post transfection, cells were treated with DMSO or 5 µM 2-thio-6-azauridine for 24 h. BRET ratios were measured as described in “Methods”. BRET ratios are normalized relative to those in control cells that were treated with DMSO (*left panel*). **b** 293T cells were co-transfected with pVphu, pcDNA-HOS-HA and pcDNA-BST-2-FLAG which express Vpu, HA tagged β-TrCP2 and Flag tagged BST-2 respectively. 24 h post transfection, cells were treated with DMSO and 5 µM 2-thio-6-azauridine for 24 h. Lysates were immunoprecipitated with 1 µg mouse anti-HA antibody followed by immunoblotting with rabbit anti-HA (*top left panel*) and Vpu antibody (*bottom left panel*). The pre-IP lysates represent 1 % of the IP input and were also immunoblotted for β-actin as a loading control (*bottom right panel*). **c** Effect of 2-thio-6-azauridine on the interaction between Vpu and BST-2. 293T cells were co-transfected with pRluc-C3-BST-2 and pEYFP-N1-Vpu, or pEYFP-N1-Vpu (S52/56N), which express Rluc tagged BST-2, EYFP tagged Vpu and EYFP tagged Vpu (S52/56N) respectively. 24 h post transfection, cells were treated with DMSO and 5 µM 2-thio-6-azauridine for 24 h. BRET ratios were measured as described in “Methods”. Values presented here are the normalized BRET ratio relative to that treated with DMSO. The *bar graphs* represent the means of results of experiments performed at least three times, and the error bars represent standard deviations. **d** 293T cells were co-transfected with pVphu, pBST-2 (1:1). 24 h post transfection, cells were treated with DMSO and 5 µM 2-thio-6-azauridine for 24 h. Lysates were immunoprecipitated with 1 µg mouse anti-HA antibody followed by immunoblotting with BST-2 (*top left panel*) and Vpu antibodies (*bottom left panel*). The pre-IP lysates represent 1 % of the IP input and were also immunoblotted for β-actin as a loading control (*bottom right panel*)

It is known that Vpu interacts with β-TrCP2, which is required for Vpu to down-regulate both CD4 and BST-2. We first established a BRET2 assay to monitor Vpu and β-TrCP2 interaction. Vpu and β-TrCP2 were fused with EYFP and RLuc, respectively. The results of Fig. 7a showed strong interaction of these two proteins and that this interaction was not affected by 2-thio-6-azauridine. This observation was further confirmed by the results of co-immunoprecipitation. 293T cells were co-transfected with plasmids expressing Vpu and HA-tagged β-TrCP2. The cells were treated with DMSO or 2-thio-6-azauridine 24 h post transfection. Cell lysates were immunoprecipitated with HA antibody and detected by immunoblotting with antibody against Vpu. The result showed that 2-thio-6-azauridine did not affect the amount of Vpu bound to β-TrCP2 (Fig. 7b).

We next examined the effect of 2-thio-6-azauridine on the interaction between Vpu and BST2. In order to avoid the interference by Vpu-mediated BST-2 degradation, we also utilized a Vpu mutant Vpu (S52/56 N) that has two Serine residues mutated at positions 52 and 56. This mutated Vpu does not interact with β-TrCP2, but preserves the BST-2 binding activity [45]. The results of both BRET2 and co-IP experiments showed that 2-thio-6-azauridine moderately increased the interaction of Vpu or Vpu (S52/56N) with BST-2 (Fig. 7c, d). Taken together, these data suggest that the inhibition of the compound on the down-regulation process does not result from interfering with the interaction among Vpu, BST-2 and β-TrCP2.

### 2-thio-6-azauridine reduces Vpu-mediated ubiquitination of BST-2

**Fig. 8:**
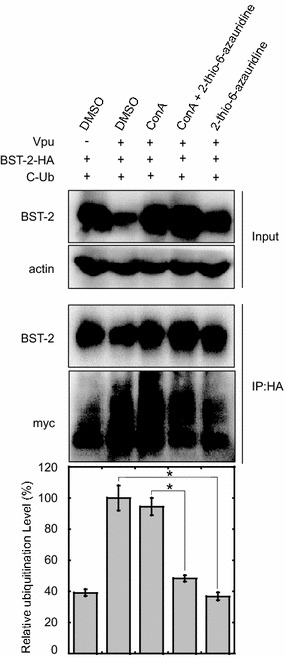
Effect of 2-thio-6-azauridine on Vpu-induced ubiquitination of BST-2. 293T cells were co-transfected with plasmid pBST-2, pVphu and CW7, which express BST-2, Vpu and C-myc tagged ubiquitin respectively (1:2:4). 24 h post transfection, cells were treated with DMSO, 5 µM 2-thio-6-azauridine and/or 50 nM Concanamycin A for another 24 h. Cell lysates were then immunoprecipitated with rabbit BST-2 antibody and treated with PNGase F, which was used to remove N-glycans from BST-2, followed by immunoblotting with mouse C-myc and HA antibody for ubiquitin and BST-2 detection respectively. The normalized ubiquitination Level of BST-2 was shown as* bar graph*

Since Vpu down-regulates BST-2 through recruiting β-TrCP2 that causes BST-2 ubiquitination, we next investigated whether 2-thio-6-azauridine exerts any effect on Vpu-mediated BST-2 ubiquitination. To this aim, we co-expressed BST-2 and Vpu in the presence or absence of Myc-tagged ubiquitin in 293T cells. At 24 h post transfection, cells were treated with DMSO, 2-thio-6-azauridine or ConA, and further cultured for another 24 h. Lysates were then immunoprecipitated with BST-2 antibody. Ubiquitin was detected by Western blotting. The results showed that 2-thio-6-azauridine obviously reduced the level of ubiquitinated BST-2, and the inhibitory effect was further enhanced in the presence of ConA that block the degradation of BST-2 (Fig. 8). These results suggest that 2-thio-6-azauridine decreases Vpu-imposed ubiquitination of BST-2.

## Discussion

In this study, we demonstrate for the first time that a small molecule compound 2-thio-6-azauridine inhibits Vpu-induced BST-2 degradation, increases BST-2 level at the cell surface in the presence of Vpu and, as a result, inhibits wild type HIV-1 infection of BST-2-expressing cells. The anti-HIV-1 property of this compound depends on its capacity to diminish Vpu-induced BST-2 ubiquitination.

2-thio-6-azauridine has been shown to inhibit RNA virus especially flaviviruses [including West Nile Virus (WNV), Yellow Fever Virus (YFV) and Japanese Encephalitis Virus (JEV)], through blocking nucleoside triphosphate synthesis [40, 41]. 2-thio-6-azauridine inhibits orotidine monophosphate decarboxylase (ODCase). It also acts as a nucleoside analog and gets incorporated into the viral RNA, which leads to impaired translation of viral RNA [40, 46, 47]. These activities of 2-thio-6-azauridine may account for the moderate inhibition of HIV-1 by this compound in the absence of BST-2 (Fig. 2). In addition, we also observed that viral production from Hela-shRNA-BST-2 was reduced by the compound to a greater extent than that from 293T cell. This might reflect different sensitivity of viral production to the inhibitory effect of 2-thio-6-azauridine in the two BST-2-deficient cell lines.

Given the potent antiviral activity of BST-2 and its constitutive expression in many human immunocytes, it is rational to devise a strategy that can expose wild type HIV-1 to this host restriction scheme. One strategy has been to identify small compounds that are able to block the BST-2 antagonist activity of HIV-1 Vpu. This strategy does not require over-expression of BST-2, which has the risk of causing cancer [48–50]. Our results show that 2-thio-6-azauridine specifically inhibits Vpu-mediated BST-2 degradation without up-regulating the expression of BST-2, nor abrogating lysosomal degradation pathway. This compound thus represents a prototype that may be further developed as a new class of anti-HIV-1 drugs that function by targeting Vpu-mediated degradation of BST-2.

Our data show that 2-thio-6-azauridine increases BST-2 levels at the cell surface without modulating the interaction between Vpu and BST-2. Rather, the level of BST-2 ubiquitination is reduced by 2-thio-6-azauridine. These data suggest that binding to BST-2 is not sufficient for Vpu to antagonize BST-2, and that this antagonization also involves Vpu-induced BST-2 ubiquitination and degradation. Despite the fact that the interaction among substrate, adaptor and E3 ligase is a prerequisite for ubiquitination and efficient removal of target protein, factors other than the physical interaction may also regulate the process. Indeed, our group as well as another have reported that the N-terminal sequences of hA3G are able to bind to Vif while remaining resistant to Vif-induced depletion by Cul5/SCF complex, suggesting binding of Vif to hA3G is required, but not sufficient for hA3G degradation [51, 52]. A recent work reported that a host protein NORE1A forms a direct complex with β-TrCP, and substrate-specifically stimulate the ubiquitin ligase activity of SCF (β-TrCP) toward its target protein β-catenin [53]. Similarly, an E3 ligase Mdm2 associate protein, p19ARF, was showed to specifically bind to Mdm2 and impairs the Mdm2-mediated degradation of p53. These observation therefore raise a possibility that 2-thio-6-azauridine might activate or inactivate a host regulator specific for Vpu-induced-ubiquitination of BST-2, without general interrupting the E3 ligase complex. Nevertheless, we also cannot exclude the possibility that pathways might exist other than β-TrCP2-dependent BST-2 degradation. For example, Janvier et al. reported that Vpu-induced BST-2 degradation may involve the Endosomal Sorting Complexes Required for Transport (ESCRT) machinery that is known to participate in sorting ubiquitinated membrane proteins toward lysosomal degradation [54].

## Conclusion

We have discovered a lead compound, 2-thio-6-azauridine, that specifically inhibits Vpu-induced BST-2 down-regulation by decreasing Vpu-mediated BST-2 ubiquitination. Given the great importance of interferon in protecting the host from virus infection, our study supports the concept of utilizing small molecule compounds to neutralize viral antagonists and thereby expose viruses to the restriction of interferon-mediated innate immune responses.

## Methods

### Plasmid DNA, antibodies and reagents

Plasmid pVphu expresses codon-optimized Vpu [55]. Plasmids pEYFP-N1-Vpu, pRluc-C3-β-TrCP2, pEYFP-N1-Vpu (S52/56N), pRluc-C3-BST-2 and pRluc-C3-β-TrCP2 for BRET2 assay were kindly provided by Dr. Chen Liang (McGill AIDS Center, Montreal, Canada). Plasmids pEYFP-N1-Vpu, pRluc-C3-β-TrCP2 and pRluc-C3-BST-2 express EYFP tagged Vpu, Rluc tagged β-TrCP2 and Rluc tagged BST-2 respectively. Plasmid pEYFP-N1-Vpu (S52/56N) expresses EYFP tagged Vpu protein with amino acid substitutions of serines for asparagines at position 52 and 56, which keeps the ability of Vpu to bind BST-2 but cannot induce its degradation. The vesicular stomatitis virus glycoprotein (VSV-G) expressing vector pHIT/G and HIV-1 proviral indicator construct pNL4-3Luc(R-E-) were kind gifts from Dr. Johnny He. Plasmid pNL-Luc-E^−^ expresses firefly luciferase reporter gene in Env-deficient HIV-1 particles, which can be rescued in one-round infection experiment by VSV-G membrane protein provided in trans by plasmid pHIT/G [56]. Plasmid pBST-2, which expresses HA tagged BST-2, was kindly provided by Dr. Paul D. Bieniasz. Plasmids pcDNA-HOS-HA and pcDNA3-IFNAR1-FLAG were generous gifts from Dr. Serge Y. Fuchs [43]. Plasmid CW7 which expresses C-myc ubiquitin was provided by Dr. Xiaofang Yu. Plasmid pcDNA3.1-K5-HA, which expresses HA tagged KSHV K5 protein, was a kind gift from Dr. Klaus Früh. pMX hCD4 expresses human CD4 (Addgene, Catalog No. 14614). Plasmid shRNA-BST-2 targeting *bst*-*2* mRNA was purchased from Sigma. Vpu and BST-2 anti-serum were obtained from National Institutes of Health (NIH) AIDS Research & Reference Reagent Program. Luciferase antibody, β-actin antibody, HRP (horseradish peroxidase)-conjugated donkey anti-rabbit and goat anti-rabbit IgG-FITC secondary antibodies were purchased from Santa Cruz Co. Concanamycin A, MG132 and doxycycline were purchased from Sigma (St. Louis, MO). Cord blood mononuclear cells (CBMC) were isolated from healthy infants after uncomplicated births. The HIV-1 proviral DNA clone HIV-1_NL4–3_ was obtained from the NIH AIDS Research and Reference Reagent Program.

### Cell culture and transfection

HeLa and 293T cells were cultured in Dulbecco’s Modified Eagle’s Medium with the addition of 10 % fetal bovine serum (FBS) (Invitrogen). HeLa-Vpu cell line was established and cultured as previously described [39]. SupT1 cells were maintained in RPMI-1640 containing 10 % FBS. 293T and HeLa cells were transfected using LipofectAMINE 2000 (Invitrogen) or Fugene HD transfection reagents (Roche) according to the manufacturer’s instructions.

### BST-2 knockdown HeLa cell line

HeLa cells were transfected with shRNA-BST-2 plasmid, then selected with 400 μg/ml G418 24 h post transfection. Limited dilution was conducted to obtain single cell colonies. One clonal cell line shRNA-BST-2 has almost no BST-2 expression and was therefore used in the subsequent experiments.

### Single-round HIV-1 replication assay

HeLa or HeLa-Vpu cells (2 × 10^5^) were co-transfected with 0.6 μg of pNL-Luc-E^−^ or pNL-Luc-E^−^-Vpu^−^ and 0.4 μg of pHIT/G. After 48 h, the viral supernatant was harvested by filtration through a 0.45 μm filter and the amount of viruses was determined by measuring the level of p24 antigen using ELISA (Biomerieux). HIV-1 of the same p24 amounts was used to infect SupT1 cells (1 × 10^5^) in 96-well plates. The SupT1 cells were lysed at 48 h post infection and the firefly luciferase activities were measured to determine the level of HIV-1 infection.

### Measuring viral reverse transcriptase activity

HIV-1 stocks were produced and quantified as previously described [42]. HIV-1 infection of primary cells followed the procedure as referenced [57]. Viral reverse transcriptase activity was measured to determine the amounts of virus in culture supernatants. Briefly, 10 μl of culture supernatant was mixed with 40 μl of reaction buffer containing 0.5 unit/ml poly(rA)-oligo(dT) (Midland Certified Reagent Co.) and 0.1 mCi/ml [^3^H]dTTP (Perkin-Elmer). After a 3-h incubation at 37 °C, reactions were terminated by the addition of 10 % trichloroacetic acid (TCA). The precipitated oligonucleotides were collected by filtering the reaction mixtures through Millipore MultiScreen Glass Fiber FC plates (Millipore). After two washes with 10 % TCA and one wash with ethanol, levels of ^3^H that were retained on the filters were scored in a liquid scintillation counter (Perkin-Elmer).

### Cell-based ELISA

HeLa or HeLa-Vpu cells were plated into 96-well plates at 1 × 10^4^ cells/well. After 48 h, the cells were washed twice with phosphate buffered saline and fixed in 4 % paraformaldehyde for 20 min at room temperature. Then cells were washed with phosphate buffered saline and incubated with 50 μl BST-2 antiserum (1:5000) for 1 h at 37 °C. Cells were washed four times with phosphate buffered saline. Next, cells were incubated with 50 μl HRP-conjugated donkey anti-rabbit secondary antibody (1:6000) for 0.5 h at 37 °C, and then washed four times with phosphate buffered saline. The cell-bound secondary antibodies were detected using 100 μl/well 3,3′,5,5′-Tetramethylbenzidine (TMB) substrate solution for 30 min at room temperature. The reactions were stopped by adding 50 μl/well 0.5 M H_2_SO_4_, followed by immediately measuring absorption at 450 nm.

### Western blotting

Cellular samples were suspended in loading buffer containing SDS and dithiothreitol and boiled for 10 min. Total proteins were separated on 12 % polyacrylamide SDS gels. Proteins were transferred onto nitrocellulose membranes and blotted with the antibodies against Vpu (1:1000), BST-2 (1:5000) or β-actin (1:1000). The membranes were further incubated with either a HRP-conjugated goat anti-mouse antibody or a HRP-conjugated donkey anti-rabbit antibody, followed by detection with enhanced chemiluminescence.

### Co-immunoprecipitation assay

293T cells were collected and lysed in 350 μl TNT buffer (20 mM Tris–HCl pH 7.5, 200 mM NaCl, 1 % Triton X-100) 48 h post transfection. The supernatants of equal amounts of protein were incubated with 3 μl of anti-HA antibody for 16 h at 4 °C, followed by the addition of protein A-Sepharose (Amersham Biosciences) for 2 h. The immunoprecipitated materials were then washed three times with TNT buffer and twice with phosphate buffered saline. After the final supernatant was removed, 30 μl of 2 × sample buffer (120 mm Tris–HCl (pH 6.8), 20 % glycerol, 4 % SDS, 2 % β-mercaptoethanol, and 0.02 % bromophenol blue) was added, and the precipitates were then boiled for 15 min to release the precipitated proteins. After centrifugation, the resulting supernatants were analyzed in Western blots.

### BRET2 assay

The BRET2 assay was used to measure protein–protein interaction. 293T cells were co-transfected with pEYFP-N1-Vpu and pRluc-C3-β-TrCP2 or with pEYFP-N1-Vpu and pBST-2. Cells were harvested 40 h after transfection, then distributed into 96-well microplates (white Optiplate; Perkin-Elmer) with a density of 2 × 10^5^ cells/well. Substrate DeepBlueC (Perkin-Elmer) was injected at a final concentration of 5 μM. The signals at 480 and 535 nm were measured sequentially on a Model 680 Microplate Reader (Berthold Technologies). BRET ratio was calculated using the equation below, [(emission at 535 nm/emission at 480 nm) in cells expressing the hRluc and hEYFP fusion proteins]—[(emission at 535 nm/emission at 475 nm) in cells expressing hRluc alone].

### Flow cytometry

Flow cytometry was performed as described previously [31]. Briefly, Cells were trypsinized and resuspended in a flow cytometry buffer (3 % fetal bovine serum in phosphate buffered saline), then stained with CD4 antiserum (1:800) and goat anti-rabbit IgG-FITC secondary antibody (1:200) on ice for 1 h, followed by fixation with 1 % paraformaldehyde and analysis on a FACS calibur system.

### Real-time RT-PCR

Total RNA was extracted from cells using TRIZOL Reagent (Invitrogen). RNA was converted to cDNA using M-MLV Reverse Transcriptase (Promega) with random primers. The cDNAs were quantified using SsoFast EvaGreen Supermix (Bio-Rad) and Bio-Rad iCycler iQ5 Real-Time PCR systems. Primer sequences for cDNAs were as follows, BST-2 sense: CTGCAACCACACTGTGATG, antisense: ACGCGTCCTGAAGCTTATG [37], GAPDH sense: GTCCACTGGCGTCTTCACCA, antisense: GTGGCAGTG ATGGCATGGAC [35]. GAPDH mRNA was quantified in order to normalize the level of *bst*-*2* mRNA.

## Additional files

10.1186/s12977-016-0247-z 2-thio-6-azauridine inhibits Vpu-mediated down-regulation of cell surface BST-2 (FACS). HeLa-Vpu and HeLa cells were treated with increasing concentrations of 2-thio-6-azauridine (0.05 µM、0.5 µM、5 µM) for 24 h. Cell surface BST-2 was measured using flow cytometry.
10.1186/s12977-016-0247-z The HIV-1 p24 value of Figure 2. (A) The HIV-1 p24 value (ng/ml) of Fig. 2A. (B) The HIV-1 p24 value (ng/ml) of 2C.
10.1186/s12977-016-0247-z FACS result of Figure 6. A) 2-thio-6-azauridine dose not affect Vpu induced down-regulation of cell surface CD4. (B) 2-thio-6-azauridine has no inhibitory effect upon K5 induced BST-2 degradation.
