# Supplementary figures and images for: 2-thio-6-azauridine inhibits Vpu mediated BST-2 degradation
Source: Retrovirology. 2016 Mar 2;13:13. doi: 10.1186/s12977-016-0247-z (PMC4776379; doi:10.1186/s12977-016-0247-z)

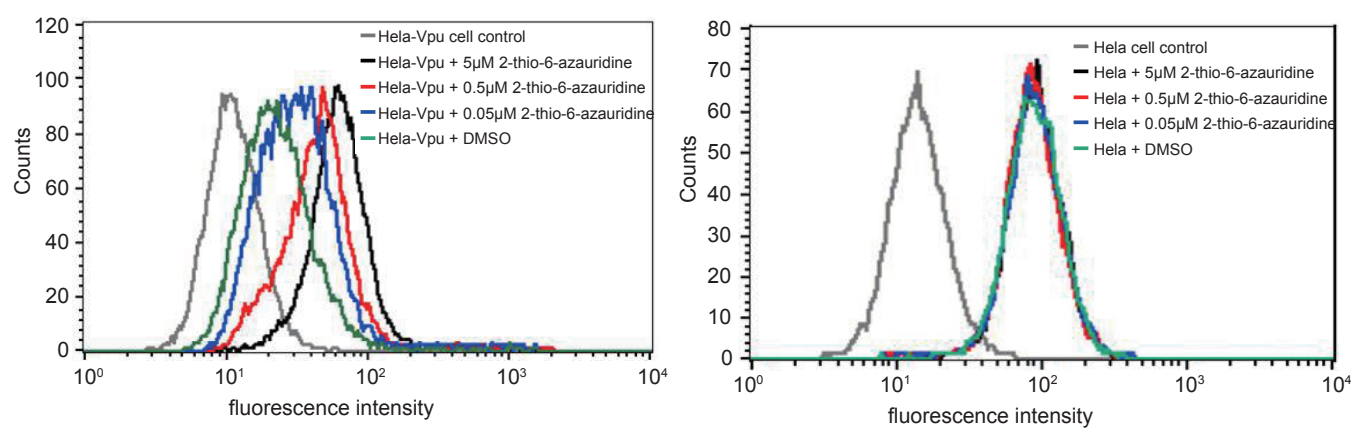

Figure S1

Supplement: Supplementary file 1 — 10.1186/s12977-016-0247-z 2-thio-6-azauridine inhibits Vpu-mediated down-regulation of cell surface BST-2 (FACS). HeLa-Vpu and HeLa cells were treated with increasing concentrations of 2-thio-6-azauridine (0.05 µM、0.5 µM、5 µM) for 24 h. Cell surface BST-2 was measured using flow cytometry. [file 12977_2016_247_MOESM1_ESM.pdf]

A

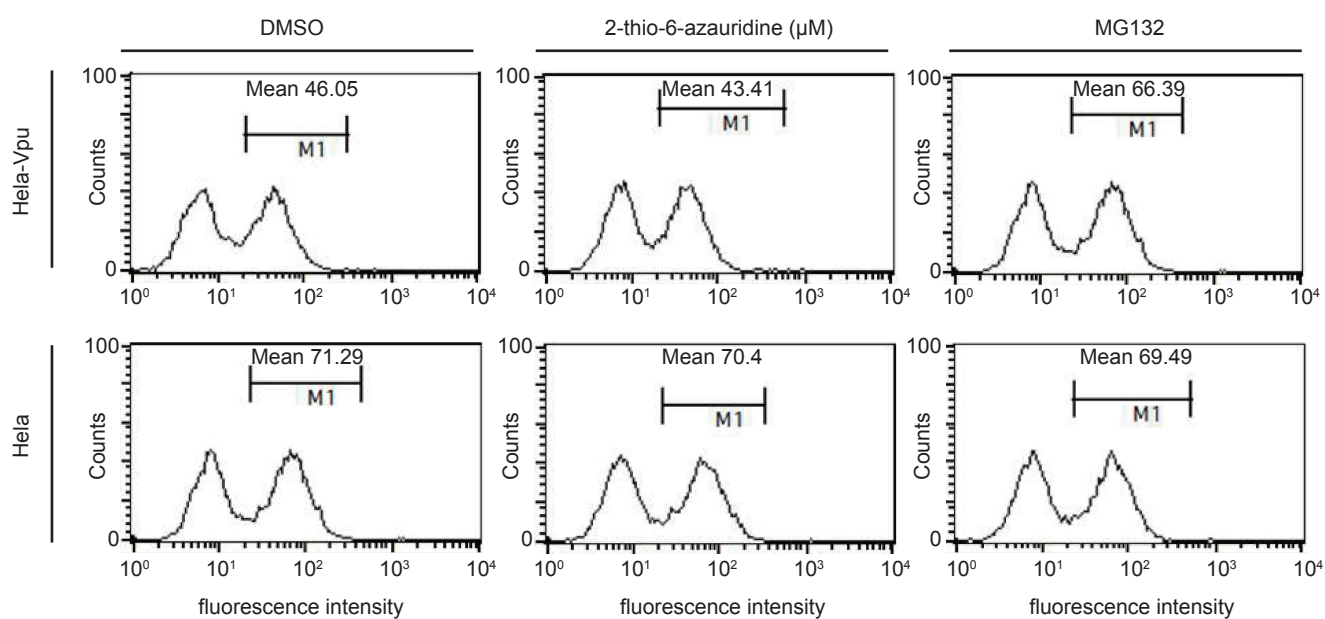

B

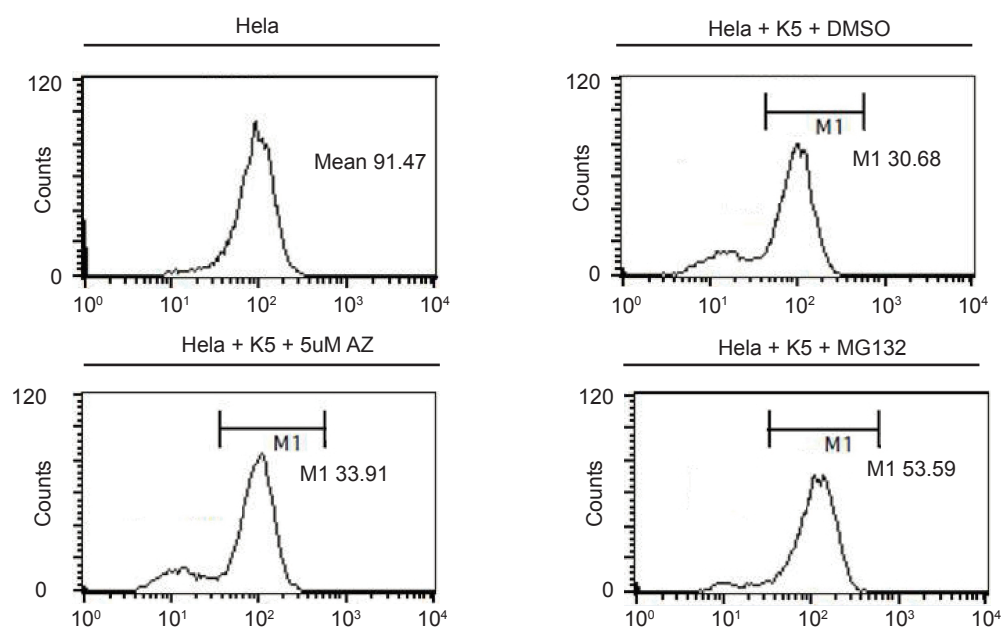

Figure S3

Supplement: Supplementary file 3 — 10.1186/s12977-016-0247-z FACS result of Figure 6. A) 2-thio-6-azauridine dose not affect Vpu induced down-regulation of cell surface CD4. (B) 2-thio-6-azauridine has no inhibitory effect upon K5 induced BST-2 degradation. [file 12977_2016_247_MOESM3_ESM.pdf]
