# Supplementary material for: 2-thio-6-azauridine inhibits Vpu mediated BST-2 degradation
Source: Retrovirology. 2016 Mar 2;13:13. doi: 10.1186/s12977-016-0247-z (PMC4776379; doi:10.1186/s12977-016-0247-z)
Supplement: Supplementary file 2 — 10.1186/s12977-016-0247-z The HIV-1 p24 value of Figure 2. (A) The HIV-1 p24 value (ng/ml) of Fig. 2A. (B) The HIV-1 p24 value (ng/ml) of 2C. [file 12977_2016_247_MOESM2_ESM.pdf]

A

number of replication	pNL-Luc-E- + pHIT/G +pCDNA3.1 +DMSO	pNL-Luc-E- + pHIT/G +pCDNA3.1 +2-thio-6-azauridine	pNL-Luc-E- + pHIT/G +BST-2 +DMSO	pNL-Luc-E- + pHIT/G +BST-2 +2-thio-6-azauridine
1	877.5	841.2	814.7	366.3
2	819.3	761.5	789.0	355.5
3	916.1	817.0	871.4	379.6

B

number of replication	Hela +DMSO	Hela + 2-thio-6-azauridine	Hela-shRNA-BST-2 +DMSO	Hela-shRNA-BST-2 + 2-thio-6-azauridine
1	291.1	108.7	606.9	374.0
2	278.3	98.7	656.9	412.7
3	320.6	95.6	695.8	429.4

Figure S2
